# Sexual Risk Reduction for HIV-Infected Persons: A Meta-Analytic Review of “Positive Prevention” Randomized Clinical Trials

**DOI:** 10.1371/journal.pone.0107652

**Published:** 2014-09-22

**Authors:** Lu Yin, Na Wang, Sten H. Vermund, Bryan E. Shepherd, Yuhua Ruan, Yiming Shao, Han-Zhu Qian

**Affiliations:** 1 Vanderbilt Institute for Global Health, Vanderbilt University School of Medicine, Nashville, Tennessee, United States of America; 2 State Key Laboratory for Infectious Disease Prevention and Control, National Center for AIDS/STD Control and Prevention, Chinese Center for Disease Control and Prevention, Beijing, China; 3 Department of Pediatrics, Vanderbilt University School of Medicine, Nashville, Tennessee, United States of America; 4 Department of Biostatistics, Vanderbilt University School of Medicine, Nashville, Tennessee, United States of America; 5 Department of Medicine, Vanderbilt University School of Medicine, Nashville, Tennessee, United States of America; 6 Collaborative Innovation Center for Diagnosis and Treatment of Infectious Diseases, Hangzhou, China; China Medical University, China

## Abstract

**Background:**

Prevention intervention trials have been conducted to reduce risk of sexual transmission among people living with HIV/AIDS (PLWHA), but the findings were inconsistent. We performed a systematic review and meta-analysis to evaluate overall efficacy of prevention interventions on unprotected vaginal or anal intercourse (UVAI) among PLWHA from randomized clinical trials (RCTs).

**Methods:**

RCTs of prevention interventions among PLWHA published as of February 2012 were identified by systematically searching thirteen electronic databases. The primary outcome was UVAI. The difference of standardized mean difference (SMD) of UVAI between study arms, defined as effect size (ES), was calculated for each study and then pooled across studies using standard meta-analysis with a random effects model.

**Results:**

Lower likelihood of UVAI was observed in the intervention arms compared with the control arms either with any sexual partners (mean ES: −0.22; 95% confidence interval [CI]: −0.32, −0.11) or with HIV-negative or unknown-status sexual partners (mean ES and 95% CI: −0.13 [−0.22, −0.04]). Short-term efficacy of interventions with ≤10 months of follow up was significant in reducing UVAI (1–5 months: −0.27 [−0.45, −0.10]; 6–10 months: −0.18 [−0.30, −0.07]), while long-term efficacy of interventions was weaker and might have been due to chance (11–15 months: −0.13 [−0.34, 0.08]; >15 months: −0.05 [−0.43, 0.32]).

**Conclusions:**

Our meta-analyses confirmed the short-term impact of prevention interventions on reducing self-reported UVAI among PLWHA irrespective of the type of sexual partner, but did not support a definite conclusion on long-term effect. It is suggested that booster intervention sessions are needed to maintain a sustainable reduction of unprotected sex among PLWHA in future risk reduction programs.

## Introduction

People who find out that they are HIV-positive may reduce their sexual and drug using behaviors, but some may continue to have difficulties with changing their risk sexual and drug using behaviors [1]. Interventions targeted towards HIV-infected individuals to reduce their risk behaviors, referred to as “positive prevention”, could be a cost-effective strategy for reducing HIV transmission [2], [3], [4]. Positive prevention has three complementary objectives in HIV-infected individuals: (1) reducing high risk sexual behaviors; (2) reducing injection and non-injection drug and alcohol abuse, where relevant; and (3) optimizing clinical care [5]. The last objective seeks to: (i) foster linkage and retention of HIV-infected persons to good care; (ii) enhance coverage and adherence to antiretroviral therapy (ART); (iii) increase full suppression of HIV viral load; and (iv) prevent development of HIV drug resistance [6], [7].

Sexual transmission, via unprotected heterosexual or homosexual contact, is the leading cause of HIV acquisition [8], [9]. Many behavioral intervention programs have been implemented for reducing unprotected sexual intercourse, but findings from these studies have been inconclusive due to heterogeneous methods [10], [11], [12], [13], [14]. Intervention components and behavioral theories have varied, populations have differed, and intensity of interventions has been heterogeneous. However, we believe it is important to assess whether across interventions, there is any overall impact of these risk reduction programs. We fully acknowledge that an intervention applied in the most strategic way in the most receptive population is optimal, but a robust prevention strategy might be revealed if diverse approaches can be demonstrated efficacious in their aggregate. A meta-analysis is warranted for the efficacy of positive prevention to generate summary outcomes from these individual studies, acknowledging their diversity in theory, method, and population, but recognizing their similarity in purpose.

An earlier meta-analysis of 12 RCTs from 1988-2004 suggested that risk reduction interventions targeting people living with HIV/AIDS (PLWHA) were efficacious in reducing 43% of self-reported unprotected sex [15]. However, several additional trials of interventions for changing sexual behaviors among PLWHA have been published in recent years [11], [12], [13], [14], [16], [17], [18], [19], [20], [21], [22], [23], [24]. Two other previous reviews of prevention interventions also reported a similar increase of condom use, but these reviews included HIV-negative individuals [25] and studies without control arms [3]. We want to provide an updated review and meta-analysis of RCTs evaluating the efficacy of prevention intervention on unprotected vaginal or anal intercourse (UVAI) among PLWHA.

## Methods

### Literature search and study selection

Our review complies with preferred reporting items for systematic reviews and meta-Analyses (PRISMA) [26], [27]. A systematic literature search was performed to identify RCTs that evaluated the impact of prevention interventions on self-reported UVAI among PLWHA, published by February 2012. Thirteen electronic databases were searched: AMED (Allied and Complementary Medicine Database, Ovid Technologies), British Library Direct, British Nursing Index (Ovid Technologies), Centre for Reviews and Dissemination databases (including DARE and NHS EED), Cochrane Library (including the Health Technology Assessment database and ENTRAL), EMBASE (Elsevier), EconLit (The American Economic Association), ERIC (Education Resources Information Centre), Ovid Medline (Ovid Technologies), PsycINFO (American Psychological Association), Scopus (Elsevier), Web of Science (Thomson Scientific Technical Support), and Global Health Library Virtual Platform (World Health Organization). Our search strategy was: (HIV-infected OR HIV-positive OR HIV-seropositive OR people living with HIV/AIDS OR AIDS OR Acquired Immunodeficiency Syndrome) AND (behavior therapy OR behavioral intervention OR risk reduction intervention OR prevention intervention OR treatment adherence OR patient compliance) AND (clinical trial OR intervention study). All publications were retrieved to an Endnote file (Endnote ×4, Thomson Reuters, San Francisco, CA), and the duplicates were deleted.

### Inclusion criteria and study selection

Studies were included if they: (1) used a randomized clinical trial (RCT) study design; (2) were HIV prevention interventions for people living with HIV/AIDS (PLWHA); (3) measured unprotected vaginal or anal intercourse; and (4) provided sufficient information to calculate effect size (ES) estimates. Studies targeting HIV-infected pregnant women were excluded because of the unique nature of antenatal care and programs to prevent mother-to-infant transmission. We did not include studies among children and young adolescents, as the interest outcome of this meta-analysis is UVAI. One trial included participants aged 16 years or older but the majority of the study sample were adults, and therefore, this study was included in our analysis [10].

All abstracts were independently reviewed by two authors (L. Yin and N. Wang), and full texts were reviewed for determining eligibility if abstracts missed key information. Papers that did not meet the above-mentioned criteria were excluded. Any disagreements were resolved by further discussion involving another author (H.-Z. Qian). The references from each eligible paper or relevant review were also examined to supplement the literature search described above, termed cross-referencing.

### Data extraction

For eligible studies, two authors extracted the following data independently in a standardized manner: authors, publication year, study period, study country, number of cities involved, approach of recruiting participants, gender distribution of participants, sample size and characteristics of participants in each study arm, possible route of acquiring HIV, description of intervention in each study arm, duration of follow-up, retention rate at the last follow-up, HIV status of sexual partners, and proportion or mean frequency of UVAI in each study arm at baseline and subsequent follow-ups. Any disagreements were reviewed and discussed by at least two authors until a consensus was reached.

### Rigor score

The rigor of study design for each of the included studies was assessed using an 8-item scale, as used in other reviews [3], [28], plus an additional item of sample size; this cut-off value of 100 for sample size item was chosen to enhance the rigor score of studies with higher statistical power. The scale is additive, with 1 point awarded for each of these 9 items. For example, if more than half of socio-demographical variables at the baseline had no statistically significant difference between study arms as shown in original studies (rigor-scale item (h)), ‘1’ was marked; otherwise ‘0’. Therefore, the rigor score can range from 0 to 9, with a higher value representing a higher rigor of study design. Note that due to our inclusion criteria of RCTs, rigor scores for all studies were 6 or greater.

### Statistical methods

The main interest of our meta-analysis was to evaluate the risk of transmitting HIV through unprotected sexual contacts among PLWHA. We analyzed the effects of prevention interventions on combined unprotected virginal and anal intercourse (UVAI) instead separately on unprotected virginal intercourse (UVI) and unprotected anal intercourse (UAI) for two reasons: (1) PLWHA in the included studies in the meta-analysis represented various risk groups such as drug users, blood donors and men who have sex with men (MSM) who could transmit HIV to male and/or female sexual partners through unprotected vaginal intercourse (UVI) or unprotected anal intercourse (UAI), (2) Most studies only reported the status of condom use, but did not specify whether using condoms during anal or virginal intercourse. All of the intervention studies that were included in the meta-analysis had measurements of UVAI at baseline and at least one follow-up time point in each study arm (i.e., intervention or control arm), other measurements were published elsewhere [29], such as alcohol use and drug use. Some studies had multiple measurements at different follow-up time points. In the latter case, the latest follow-up measurement was used in the primary meta-analysis for estimating the overall effect size; in additional subgroup analyses considering the efficacy of interventions at different time points, each follow-up measurement was considered. For studies with more than one intervention arm [10], [20], [22], [24], [30], effect sizes were calculated using the same control condition. The primary meta-analysis ignored correlation between interventions from the same study arm; the robustness of results to this correlation was assessed in sensitivity analyses that removed correlated studies. As the measurements were either expressed as proportion differences or as mean differences of frequency of UVAI, we converted estimates to a common metric of standard mean differences (SMD) using a Cox transformation [31], [32]. SMD in each study arm was calculated as the difference of mean at follow-up and baseline divided by the pooled standard deviation (SD) of these two means [33]. We contacted available authors when published articles provided insufficient information to make the calculations; four studies were excluded because insufficient data was provided by study authors [34], [35], [36], [37]. As study arms might not be comparable at baseline, even in RCTs, Becker's strategy was used to adjust for baseline UVAI between study arms [33]. The difference of SMDs between study arms, defined as effect size (ES), was calculated for each study and then pooled across studies using standard meta-analysis with a random effects model [38]. A negative value of SMD difference indicates reduction of UAVI in the intervention arm compared to the control arm. Random effect estimates allow for variation of the true effects across studies [39], and were derived using the DerSimonian-Laird method [40], [41]. The meta-analysis results were displayed with forest plots separately by type of sexual partners (any sexual partners and HIV-negative or unknown sexual partner [HNUP]).

Heterogeneities were assessed by I^2^ statistics [42], and standardized deleted residual analyses were performed to identify outliers. The funnel plot, Begg and Mazumdar rank correlation test and Egger's test of the intercept were employed to assess indications of publication bias [43].

Subgroup analyses were performed to examine effect sizes by recall period on UAVI (>3 months, 3 months, or <1 month), number of study cities (>1 or 1), participant recruitment (institution-based or non-institution-based), formats of the delivering intervention (group-based or individual-based), durations of follow-up (≥15 months, 11–15 months, 6–10 months, 1–5 months or immediately after intervention), retention rates at the latest follow-up (<80% or ≥80%), sample sizes at baseline (≤300 or>300), rigor scores (<9 or 9), and risk groups (men who have sex with men [MSM] or other population). Meta-regression was also used to examine the relationship of above-mentioned between-groups effects, except for duration of follow-ups (because outcomes at multiple follow-ups were often reported in individual studies).

Sensitivity analyses were conducted to determine the stability of the intervention efficacy estimate by evaluating whether the overall effect size was sensitive to inclusion of any individual studies. Studies excluded in iterative sensitivity analyses included those producing outliers identified by standardized deleted residuals analyses, those involving two active intervention arms contrasted to the same control arm in the same study, those that noted statistically significant efficacy on UVAI, those published before the year 2006, those targeting MSM only, and those with sample sizes less than 300 participants. All meta-analyses were performed by two authors independently in the R/S plus Software version 2.15.1 [44].

## Results

### Results of literature search

**Figure 1:**
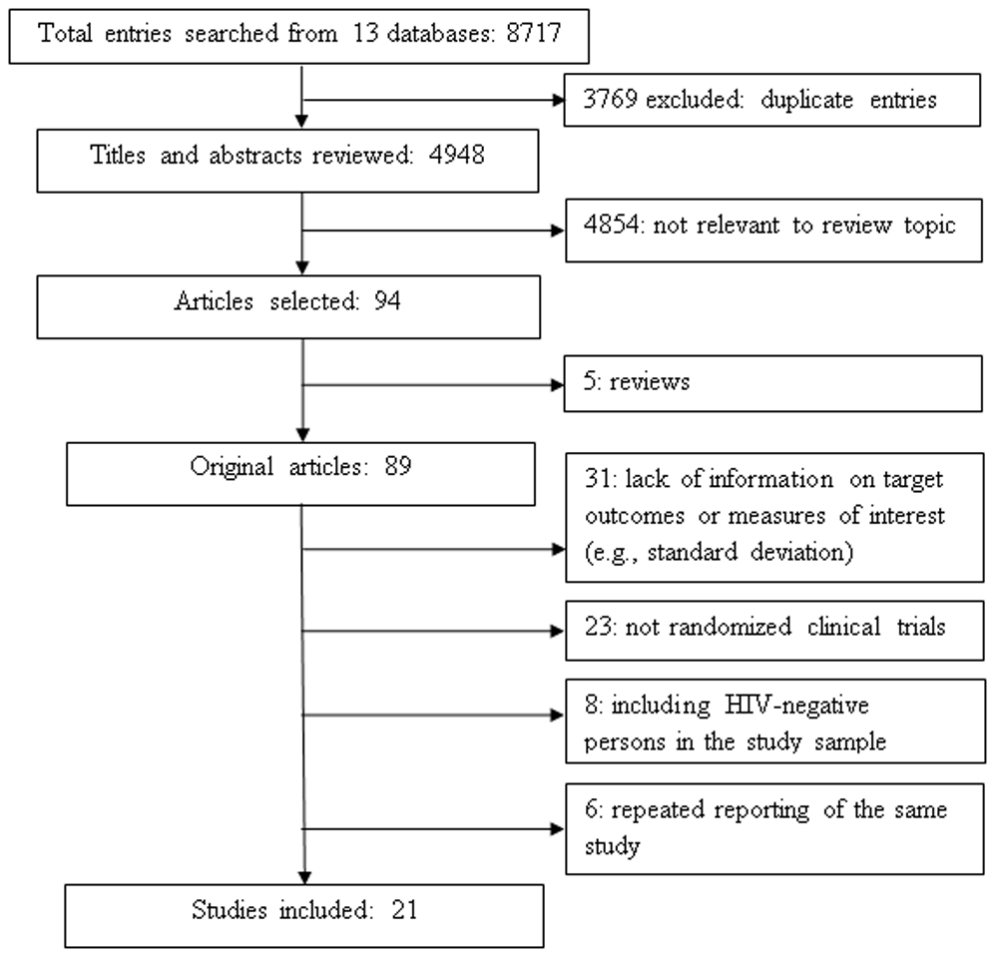
Flow diagram of literature search process^1^. ^1^ Thirteen databases included: 1) AMED; 2) British Library Direct; 3) British Nursing Index; 4) Centre for Reviews and Dissemination databases; 5) Cochrane Library; 6) EMBASE; 7) EconLit; 8) ERIC; 9) Ovid Medline; 10) PsycINFO; 11) Scopus; 12) Web of Science; and (13) Global Health Library Virtual Platform (World Health Organization).

The initial searches in 13 individual electronic databases yielded 8717 entries meeting our predefined inclusion criteria, of which 3769 were duplicate and were excluded (Figure 1). A total of 4948 titles and abstracts were reviewed, and 4854 were excluded because they were deemed irrelevant to the review topic. In the remaining 94 articles, 73 were further excluded for the following reasons: not original articles but rather editorials, comments or reviews (k = 5); lack of information on target outcome or measure of interest (k = 31); not RCTs (k = 23); HIV-negative study subjects included (k = 8); and repeated reporting of the same study (k = 6). These 73 excluded studies are listed in the supplementary material (File S1). Finally, 21 citations were included in the meta-analysis [10], [11], [12], [13], [14], [16], [17], [18], [19], [20], [21], [22], [23], [24], [30], [45], [46], [47], [48], [49], [50].

### Description of studies

All of the 21 included studies were conducted in the United States (Table 1). The sample sizes ranged from 51 to 3,556 at baseline and totaled 11,286 PLWHA. In most studies (k = 18), the participants were recruited by AIDS-service-organization-based sampling (ASOBS), such as from hospitals, clinics, or detoxification centers. Duration of interventions lasted from 3 to 18 months. The retention rates of participants ranged between 30% and 95%. The commonly used behavioral theories included information-motivation-behavioral skills model [10], [11], [14], [16], [17], [20], [21], [24], [30], [47], [50], social cognitive theory/social learning theory [11], [19], [45], [46], [49], [50], cognitive-behavioral coping [10], [18], [47], and theory of planned behavior [10], [50]. The rigor score of included studies ranged from 6 to 9 with a mean score of 7.95. Eight RCTs had a full score of 9 [11], [12], [17], [19], [21], [23], [49], [50] (Table 2).

**Table 1:** Randomized clinical trials of prevention intervention among PLWHA^1^^,^ 2.

Publication	Country (trial period)	No. of cities		Study participants					Description of interventions			Duration of follow-up (months)	Retention (%)
				Venue of recruiting participants	Gender(%)	Risk group	No. of participants (Mean and range of age at baseline)		IG		CG		
							IG	CG					
Cleary et al. [45], 1995	USA (1986–1989)	1		ASOBS	78% Male	Blood donors	Pre-intervention 135 Post-intervention 94 (32, 18–55+)	Pre-intervention 136 Post-intervention 106 (32, 18–55+)	Cognitive behavioral and skills training support group		Community referral program	12	IG: 70 CG: 78
Kalichman et al. [46], 2001	USA (N/A)	1		ABS, ASOBS	70% Male	89% MSM	Pre-intervention 185 Post-intervention 150F3 or 146F6 (40, N/A)	Pre-intervention 143 Post-intervention 121F3 or 110F6 (40, N/A)	Group intervention focused on strategies for practicing safer sexual behavior		Contact-matched, health-maintenance support group	6	IG: 79 CG: 77
Margolin et al. [47], 2003	USA (1997–2001)	1		ASOBS	70% Male	IDU	Pre-intervention 45 Post-intervention 32F0 or 34F3 (41, N/A)	Pre-intervention 45 Post-intervention 32F0 or 29F3 (41, N/A)	Manual-Guided HIV+ harm reduction program		Enhanced methadone maintenance program	3	82
Sorensen et al. [48], 2003	USA (1994–1998)	1		ASOBS	73% Male	DU	Pre-intervention 92 Post-intervention 77F6, 73F12, 73F18 (39, N/A)	Pre-intervention 98 Post-intervention 82F6, 77F12, 77F18 (38, N/A)	Case management delivered by paraprofessionals		A brief contact condition	18	79
Richardson et al. [30], 2004	USA (1998–2001)	1 state		ASOBS	86% Male	74% MSM	Pre-intervention 265^ A^ or 324^B^ Post-intervention 175^ A^ or 214^B^ (38, ≥18)	Pre-intervention 297 Post-intervention 196 (39, ≥18)	A: gain-frame: positive consequences of safer sex B: loss-frame: negative consequences of unsafe sex		Attention-control: medication adherence	7	66
Rotheram-Borus et al. [10], 2004	USA (1999–2004)	3		ASOBS, ABS, RDS, CBS	78% Male	69% MSM	Pre-intervention 59^C^ or 61^D^ Post-intervention 48^C^ or 50^D^ (23, 16–29)	Pre-intervention 55 Post-intervention 45 (23, 16–29)	C: telephone delivered intervention D: in-person delivered intervention		Delayed-intervention	15	82
Wingood et al. [49], 2004	USA (1997–2001)	2 states		ASOBS	Female	SAA	Pre-intervention 190 Post-intervention 176F6 or 162F12 (34, 18–50)	Pre-intervention 176 Post-intervention 165F6 or 159F12 (34, 18–50)	Sexual risk reduction and social networks	Adherence and nutrition		12	IG: 85 CG: 90
Wolitski et al. [50], 2005	USA (2000–2002)	2		CBS	Male	MSM	Pre-intervention 413 Post-intervention 358F3 or 375F6 (41, 20–89)	Pre-intervention 398 Post-intervention 335F3 or 354F6 (41, 20–89)	Enhanced intervention (safer sex information, learning activities, peer-led discussion groups)	Standard intervention (safer sex information)		6	IG: 91 CG: 89
Naar-King et al. [16], 2006	USA (N/A)	1		ASOBS	51% Male	41% MSM	Pre-intervention 25 Post-intervention 21 (21, 16–25)	Pre-intervention 26 Post-intervention 26 (21, 16–25)	Four-session motivational enhancement intervention	Wait-list control intervention		6	80
Purcell et al. [11], 2007	USA (2001–2005)	4		CBS	60% Male	IDU	Pre-intervention 486 Post-intervention 419F3, 402F6, 417F12 (42, 22–60)	Pre-intervention 480 Post-intervention 421F3, 404F6, 404F12 (42, 22–60)	Peer mentoring intervention	Video discussion intervention		12	IG: 86 CG: 84
Gilbert et al. [17], 2008	USA (2003–2006)	1		ASOBS	79% Male	51% MSM	Pre-intervention 240 Post-intervention 182F3 or 200F6 (43, ≥18)	Pre-intervention 231 Post-intervention 188F3 or 193F6 (44, ≥18)	Tailored risk-reduction counseling from a “Video Doctor”	Usual care		6	IG: 83 CG: 84
Sikkema et al. [18], 2008	USA (2002–2005)	1		CBS, ASOBS	48% Male	People with CSA histories	Pre-intervention 102 Post-intervention 88F0, 88F4, 84F8, 81F12 (41, ≥18)	Pre-intervention 104 Post-intervention 95F0, 88F4, 87F8, 82F12 (42, ≥18)	HIV and trauma coping group experimental intervention	Time-matched HIV support group comparison		12	IG: 79 CG: 79
Williams et al. [19], 2008	USA (2003–2006)	1		ABS, ASOBS	Male	People with CSA histories	Pre-intervention 75 Post-intervention 70 (43, ≥18)	Pre-intervention 62 Post-intervention 58 (43, ≥18)	Sexual health intervention for men guided by cognitive-behavioral approaches	Standard health promotion Intervention		6	93
McKirnan et al. [12], 2010	USA (2004–2006)		1	ASOBS	Male	MSM	Pre-intervention 166 Post-intervention 133F6 or 152F12(42, 18–50+)	Pre-intervention 151 Post-intervention 122F6 or 139F12 (42, 18–50+)	Peer-led treatment advocacy program, primary-care-based, individual counseling intervention	Standard care		12	IG: 92 CG: 94
Myers et al. [20], 2010	USA (2004–2007)		13	ASOBS	70% Male	52% MSM	Pre-intervention 768^ E^, 975^ F^, 758^ G^ Post-intervention 583^ E^, 604^ F^, 333^ G^ (N/A, ≥18)	Pre-intervention 1055 Post-intervention 612 (N/A, ≥18)	E: MCP-delivered intervention F: PS-delivered intervention G: interventions delivered by MCP and PS	Standard care		12	IG: 76^A^ 62^B^ 44^C^ CG: 58
Rose et al. [21], 2010	USA (2004–2006)		1	ASOBS	69% Male	SAA	Pre-intervention 181 Post-intervention 161 (43, 25–65)	Pre-intervention 205 Post-intervention 167 (43, 25–65)	HIV risk-reduction intervention	Standard care		6	IG: 89 CG: 81
Rosser et al. [22], 2010	USA (2005–2007)		6	ABS, CBS, ASOBS, WBS	Male	MSM	Pre-intervention 248^H^ or 237^I^ Post-intervention 197F6 ^,H^, 194F6 ^,I^ 196F12 ^,H^,185F12 ^,I^ 188F18 ^,H^, 184F18 ^,I^ (N/A, 18–45+)	Pre-intervention 190 Post-intervention 166F6,161F12,155F18 (N/A, 18–45+)	H: Man to Man sexual health seminar; I: positive tailored sexual health seminar	Prevention video (men speaking out)		18	IG: 76^A^ 78^B^ CG: 82
Teti et al. [13], 2010	USA (2004–2007)		1	ASOBS, PRS, ABS	Female	86% Black adults	Pre-intervention 92 Post-intervention 61F6, 46F12, 28F18 (40, 20–70)	Pre-intervention 92 Post-intervention 70F6, 52F12, 27F18 (38, 20–70)	Received messages, group-level, peer-led support intervention	Received brief messages		18	IG: 30 CG: 29
Wolitski et al. [23], 2010	USA (2004–2007)		3	ASOBS	70% Male	Homeless and unstably housed adults	Pre-intervention 315 Post-intervention 301F6, 284F12, 274F18 (N/A, 18–50+)	Pre-intervention 315 Post-intervention 275F6, 266F12, 259F18 (N/A, 18–50+)	Immediate housing opportunities for people with AIDS rental assistance	Customary housing service		18	IG: 87 CG: 82
Lovejoy et al. [24], 2011	USA (2009–2010)		5	ABS, ASOBS	54% Male	Middle-age and older adults	Pre-intervention 38^ J^ or 39^K^ Post-intervention 34F3 ^,J^,36 F6 ^,K^ 36F3 ^,J^, 36 F6 ^,K^ (53, 45–66)	Pre-intervention 23 Post-intervention 22F3,23F6 (53, 45–66)	J: 4-session TDMII K: 1-session TDMII	No		6	IG: 95^A^ 92^B^ CG: 100
Golin et al. [14], 2012	USA (2006–2009)		2	ASOBS	65% Male	SAA	Pre-intervention 248 Post-intervention 206F4, 183F8, 154F12 (43, ≥18)	Pre-intervention 242 Post-intervention 206F4, 187F8, 155F12 (43, ≥18)	SafeTalk: 4-session MI-based safer sex program	New Leaf: 4-session heart-healthy attention-matched control program		12	63

1 PLWHA: people living with HIV/AIDS; IG: intervention group; CG: control groups; ABS: advertisement-based sampling; CBS: community-based sampling; ASOBS: AIDS-service-organization-based sampling; PRS: peer-referral sampling; RDS: response-driven sampling; WBS: web-based sampling; MSM: men who have sex with men; DU: drug users; IDU: injection drug users; CSA: child sexual abuse; SAA: sexually active adults; MCP: medical care provider; PS: prevention specialist; TDMII: telephone-delivered motivational interviewing interventions; N/A: not available;

2 Some studies explored 2–3 intervention groups, including A: gain-frame positive consequences of safer sex; B: loss-frame, negative consequences of unsafe sex; C: telephone delivered intervention; D: in-person delivered intervention; E: medical care provider-delivered intervention; F: prevention specialist-delivered intervention; G: interventions delivered by primary care providers and prevention specialists; H: Man to Man sexual health seminar; I: positive tailored sexual health seminar; J: 4-session telephone-delivered motivational interviewing intervention; K: 1-session telephone-delivered motivational interviewing intervention;

F0 Immediately after intervention;

F3 In the 3-month follow-up;

F4 In the 4-month follow-up;

F6 In the 6-month follow-up;

F8 In the 8-month follow-up;

F12 In the 12-month follow-up;

F18 In the 18-month follow-up.

**Table 2:** Rigor score of study design^1^.

Publication	Cohort(a)	With control group (b)	Pre/post intervention(c)	Random assignment(d)	Random selection for assessment (e)	Sample size >100 (f)	Follow-up rate ≥80%(g)	Comparable socio-demographics between study arms (h)	Comparable baseline outcome measures between study arms (i)	Total
Cleary et al. [45], 1995	1	1	1	1	1	1	0	1	0	7
Kalichman et al. [46], 2001	1	1	1	1	1	1	0	1	1	8
Margolin et al. [47], 2003	1	1	1	1	1	0	1	1	0	7
Sorensen et al. [48], 2003	1	1	1	1	1	1	0	1	0	7
Richardson et al. [30], 2004	1	1	1	1	1	1	0	0	0	6
Rotheram-Borus et al. [10], 2004	1	1	1	1	1	1	1	1	0	8
Wingood et al. [49], 2004	1	1	1	1	1	1	1	1	1	9
Wolitski et al. [50], 2005	1	1	1	1	1	1	1	1	1	9
Naar-King et al. [16], 2006	1	1	1	1	1	0	1	1	1	8
Purcell et al. [11], 2007	1	1	1	1	1	1	1	1	1	9
Gilbert et al. [17], 2008	1	1	1	1	1	1	1	1	1	9
Sikkema et al. [18], 2008	1	1	1	1	1	1	0	1	1	8
Williams et al. [19], 2008	1	1	1	1	1	1	1	1	1	9
McKirnan et al. [12], 2010	1	1	1	1	1	1	1	1	1	9
Myers et al. [20], 2010	1	1	1	1	1	1	0	0	0	6
Rose et al. [21], 2010	1	1	1	1	1	1	1	1	1	9
Rosser et al. [22], 2010	1	1	1	1	1	1	0	1	1	8
Teti et al. [13], 2010	1	1	1	1	1	1	0	1	0	7
Wolitski et al. [23], 2010	1	1	1	1	1	1	1	1	1	9
Lovejoy et al. [24], 2011	1	1	1	1	1	0	1	1	0	7
Golin et al. [14], 2012	1	1	1	1	1	1	0	1	1	8

1 1-point score was given for meeting each of the following criteria: (a) being a prospective cohort study; (b) using a comparison group; (c) collecting pre-/post-intervention data; (d) employing random assignment of participants to study arms; (e) having all study participants or a random selection of them for assessments; (f) having a sample size >100; (g) having a follow-up rate ≥80%; (h) having comparable socio-demographics between study arms, including age, education, race, employment, income, marital status (“Comparability” was defined as more than half of socio-demographical variables had no statistically significant difference between study arms as shown in the original publication, and ‘1’ was marked; otherwise ‘0’); and (i) had comparable outcome measures at baseline between study arms.

### Impact of prevention intervention on UVAI with any sexual partners

Eight studies provided mean frequencies of UVAI [14], [16], [18], [19], [24], [46], [48], [49], and 13 provided proportions of UVAI [10], [11], [12], [13], [17], [20], [21], [22], [23], [30], [45], [47], [50]. Reduction of UVAI was observed in the intervention arm in most studies, except that two studies observed an increased risk of UVAI in the gain-framed approach or telephone-delivered intervention measures [10], [30]. Of these 21 trials, 15 reported UVAI with any sexual partners, and 10 reported UVAI with HNUP.

**Figure 2:**
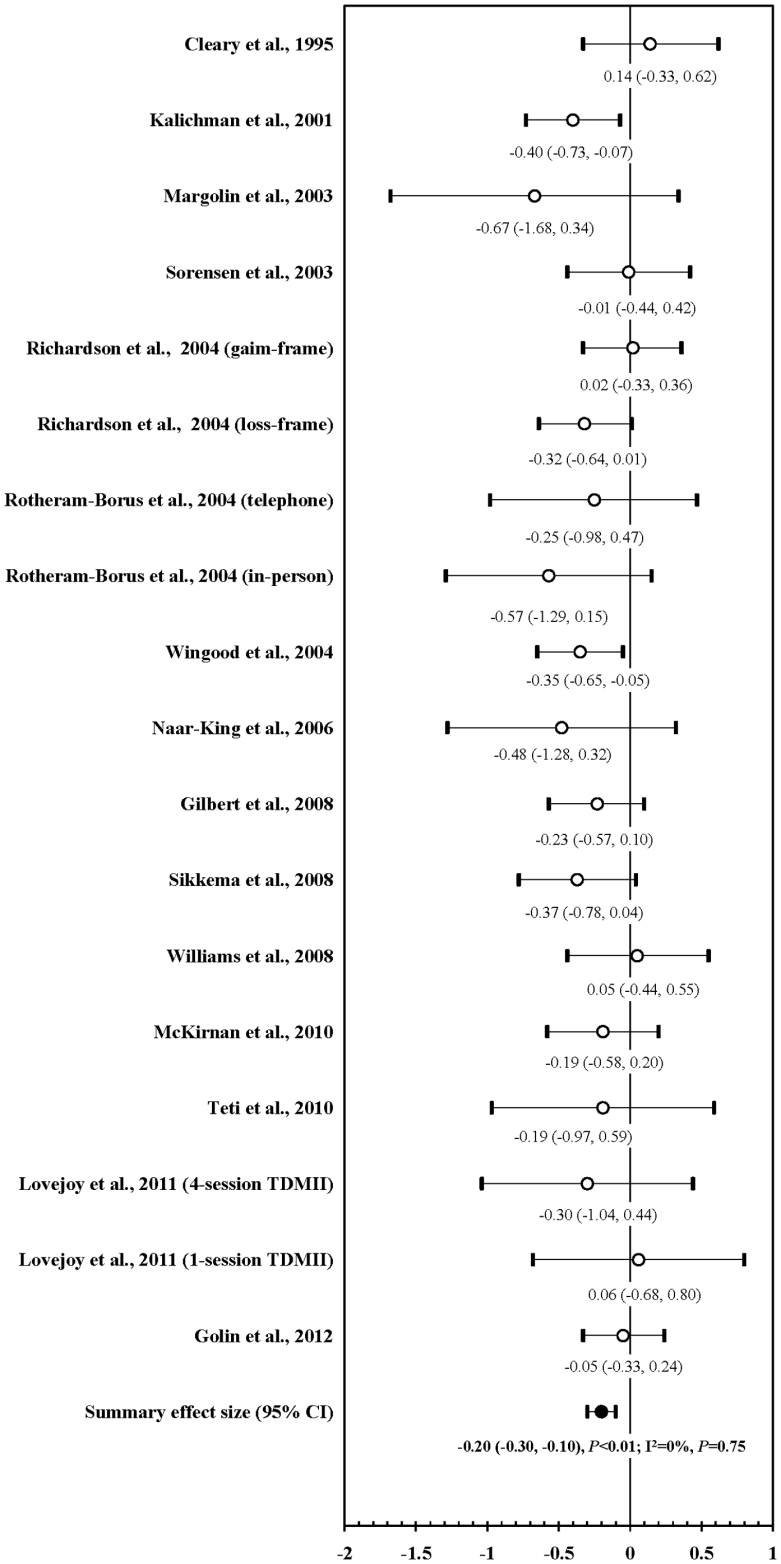
Forest plot of effect size: impact of prevention interventions on UVAI with any sexual partners among PLWHA^1^. ^1^ UVAI: unprotected vaginal and/or anal intercourse; PLWHA: people living with HIV/AIDS; TDMII: telephone-delivered motivational interviewing interventions.

Figure 2 shows the efficacy of prevention interventions on UVAI with any sexual partners from 18 interventions in 15 RCTs. Of them, 14 intervention measures showed that prevention interventions reduced UVAI after adjusting for the baseline difference between study arms, but only two were statistically significant [46], [49]. Four intervention measures observed an increased risk in intervention arms versus control arms [19], [24], [30], [45]. After pooling, the meta-analysis demonstrated a lower average UVAI with any sexual partners in the intervention arms compared with the control arms (mean ES: −0.20; 95% CI: −0.30, −0.10; *P*<0.01; k = 15). There was null heterogeneity among these 15 studies (I^2^ = 0%; *P* = 0.75). The funnel plot showed no evidence of publication bias (Kendall tau = −0.05; *P* = 0.82; Egger's t value = −0.41; *P* = 0.68).

Table 3 summarizes the subgroup analyses of the efficacy of intervention on UVAI with any sexual partners. On average, the interventions reduced UVAI with any sexual partner within 10 months post intervention (or “short-term” effect) (mean ES [95% CI]: 1–5 months: −0.23 [−0.37, −0.08], k = 8; 6–10 months: −0.18 [−0.29, −0.08], k = 14), while the long-term impact of the interventions was not statistically significant (mean ES [95% CI]: 11–15 months: −0.12 [−0.30, 0.06], k = 9; >15 months: −0.05 [−0.43, 0.32], k = 2). A reduction of UVAI was observed among studies in which UVAI was recalled in the past 3 months (mean ES [95% CI]: −0.21 [−0.34, −0.08], k = 10), but not among studies where UVAI was recalled for ≤1 month or >3 months (both k = 4). Group-based interventions appeared more effective (mean ES [95% CI]: −0.27 [−0.42, −0.12], k = 12) than individual-based interventions (−0.13 [−0.27, 0.01], k = 6). RCTs with a sample size of >300 showed a reduction of UVAI (mean ES [95% CI]: −0.26 [−0.34, −0.09], k = 7), but those with a sample size of ≤300 did not reach statistical significance (mean ES [95% CI]: −0.16 [−0.34, 0.02], k = 11). Prevention intervention effects were not substantially affected by number of study cities, approach of recruiting participants, retention rate at the last follow-up, and rigor score of study design. With the above noted, it is important to point out that in meta-regression, none of these factors statistically modified the overall effect size of UVAI with any sexual partners (P>0.05).

**Table 3:** Subgroup and sensitivity analyses for UVAI with any sexual partners among PLWHA^1^.

Subgroup	No. of intervention	Combined effect size (95% CI)	*P* value	Heterogeneity	
				I^2^ (%)	*P* value
Recall period on UVAI (months)					
≤1	4	−0.16 (−0.44, 0.13)	0.28	33.4	0.21
3	10	−0.21 (−0.34, −0.08)	<0.01	0	0.68
>3	4	−0.19 (−0.43, 0.04)	0.11	0	0.65
Number of study cities					
1	10	−0.21 (−0.35, −0.06)	0.01	0	0.62
>1	8	−0.19 (−0.33, −0.05)	0.01	0	0.59
Venue of recruiting participants					
AIDS service organizations (ASO)	10	−0.17 (−0.29, −0.05)	0.01	0	0.55
Non-ASO	8	−0.28 (−0.47, −0.09)	<0.01	0	0.79
Format of delivering intervention					
Group-based	12	−0.27 (−0.42, −0.12)	<0.01	0	0.71
Individual-based	6	−0.13 (−0.27, 0.01)	0.06	0	0.69
Duration of follow-up (months)					
Immediately after intervention	3	−0.27 (−0.56, 0.03)	0.07	0	0.82
1–5	8	−0.23 (−0.37, −0.08)	<0.01	0	0.92
6–10	14	−0.18 (−0.29, −0.08)	<0.01	0	0.81
11–15	9	−0.12 (−0.30, 0.06)	0.18	32.7	0.16
>15	2	−0.05 (−0.43, 0.32)	0.78	0	0.69
Retention rate at the last follow-up					
<80%	8	−0.16 (−0.30, −0.02)	0.03	7.5	0.37
≥80%	10	−0.26 (−0.41, −0.10)	<0.01	0	0.89
Sample size at baseline					
≤300	11	−0.16 (−0.34, 0.02)	0.08	0	0.71
>300	7	−0.26 (−0.34, −0.09)	<0.01	0	0.50
Publication year					
Prior to 2006	9	−0.22 (−0.37, −0.08)	<0.01	9.6	0.36
In 2006 or later	9	−0.17 (−0.31, −0.02)	0.03	0	0.89
Rigor score					
<9	14	−0.19 (−0.31, −0.06)	<0.01	0	0.63
9	4	−0.23 (−0.41, −0.05)	0.01	0	0.60
Sensitivity analyses					
Excluded Cleary et al. [45], 1995	17	−0.22 (−0.32, −0.11)	<0.01	0	0.83
Excluded Kalichman et al. [46], 2001	17	−0.18 (−0.28, −0.07)	<0.01	0	0.79
Excluded Richardson et al. [30], 2004 (gain-frame)	17	−0.22 (−0.33, −0.11)	<0.01	0	0.80
Excluded Richardson et al. [30], 2004 (loss-frame)	17	−0.19 (−0.29, −0.08)	<0.01	0	0.73
Excluded Rotheram-Borus et al. [10], 2004 (telephone)	17	−0.20 (−0.30, −0.10)	<0.01	0	0.69
Excluded Rotheram-Borus et al. [10], 2004 (in-person)	17	−0.19 (−0.29, −0.09)	<0.01	0	0.76
Excluded Wingood et al. [49], 2004	17	−0.18 (−0.29, −0.07)	<0.01	0	0.76
Excluded Naar-King et al. [16], 2006	17	−0.19 (−0.30, −0.09)	<0.01	0	0.72
Excluded McKirnan et al. [12], 2010	17	−0.20 (−0.30, −0.09)	<0.01	0	0.69
Excluded Lovejoy et al. [24], 2011 (4-session TDMII)	17	−0.20 (−0.31, −0.10)	<0.01	0	0.72
Excluded Lovejoy et al. [24], 2011 (1-session TDMII)	17	−0.20 (−0.30, −0.10)	<0.01	0	0.69

1 UVAI: unprotected vaginal or anal intercourse; PLWHA: people living with HIV/AIDS; TDMII: telephone-delivered motivational interviewing interventions.

In standardized deleted residual analysis, no individual study was identified as an outlier, but Teti et al. 's study [13] was found as an outlier in the subgroup of 11–15 months follow-up (standardized deleted residual  = 2.04). Further sensitivity analyses were used to evaluate the stability of summary effect sizes in meta-analyses for these considerations: use of multiple interventions in a single trial [10], [24], [30], statistically significant efficacy on UVAI [46], [49], publication prior to year 2000 [45], targeting of MSM only [12], and sample size less than 100 [16]. After excluding any one of the above-mentioned studies or interventions, the statistically significant association between interventions and UVAI was still observed (Table 3). Additional sensitivity analysis was performed to determine the stability of the intervention efficacy in the subgroup of 11–15 months follow-up, and found a significant reduction of UVAI during 11–15 months follow-up after intervention (mean ES [95% CI]: −0.17 [−0.32, −0.02; P = 0.03]; I^2^ = 7.4%, P = 0.37; k = 8).

### Impact of prevention intervention on UVAI with HNUP

**Figure 3:**
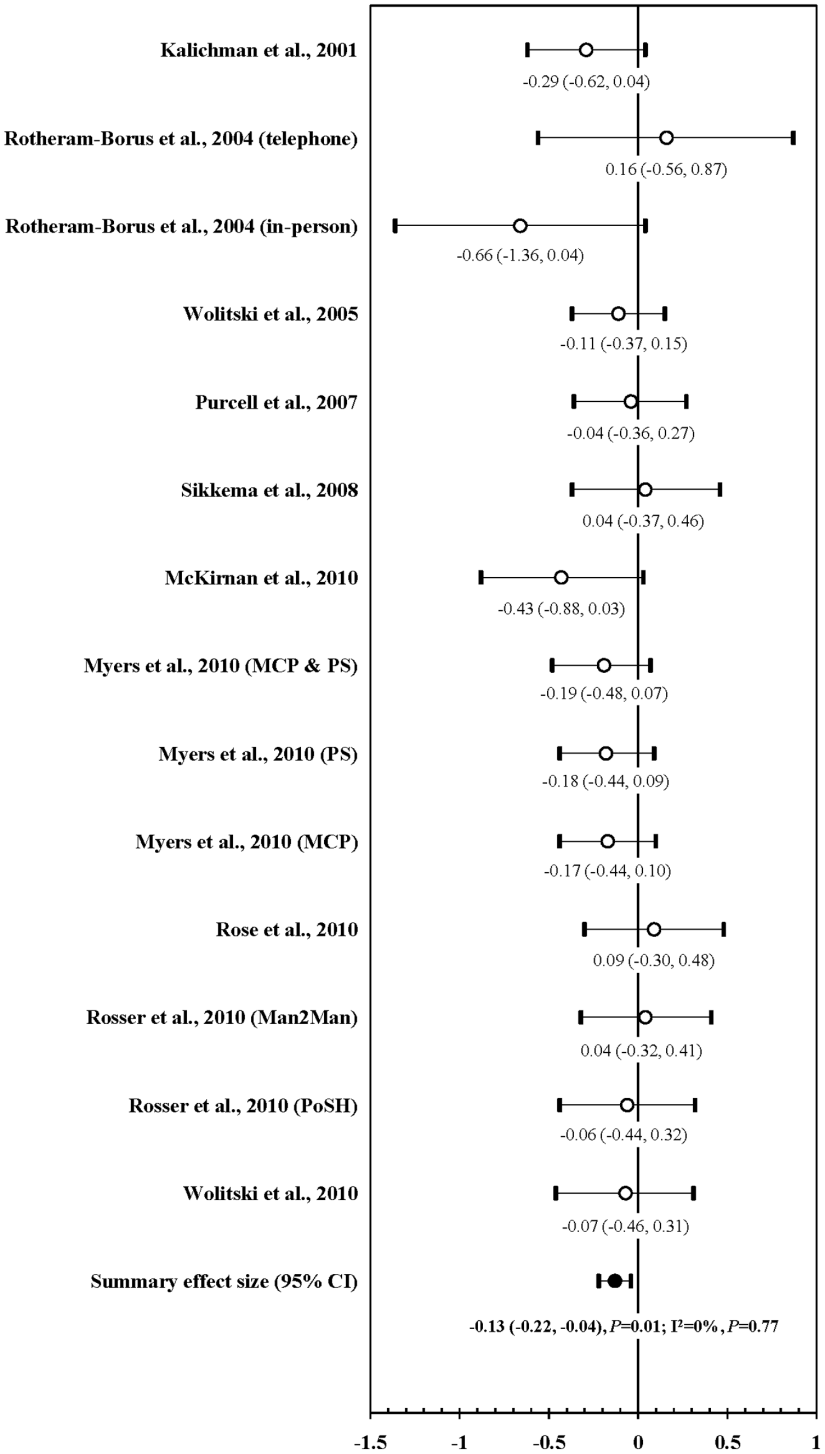
Forest plot of effect size: impact of prevention interventions on UVAI with HIV negative or unknown status sexual partner among PLWHA^1^. ^1^ UVAI: unprotected vaginal and/or anal intercourse; PLWHA: people living with HIV/AIDS; MCP: medical care provider; PS: prevention specialist; Man2Man: man to man sexual health seminar; PoSH: positive sexual health seminar.

Ten RCTs including 14 interventions evaluated the impact of interventions on UVAI with HNUP. Among these RCTs, ten intervention measures had a reduction of UVAI and four increased UVAI, but none reached statistical significance. However, a meta-analysis showed that prevention interventions were associated with a significant average reduction of UVAI with HNUP (mean ES: −0.13; 95% CI: −0.22, −0.04; *P* = 0.01). Neither heterogeneity (I^2^ = 0%; *P* = 0.77) nor publication bias were observed (Kendall tau  = 0.14; *P* = 0.52; Egger's t value  = −0.004; *P* = 0.997) (Figure 3).

Similar subgroup analyses were also conducted for UVAI with HNUP as were for UVAI with any sexual partners. The efficacy of interventions was statistically significant only among some subgroups of interventions: those with the recall period on UVAI >3 months (*P* = 0.02), those with the number of study cities >1 (*P* = 0.02), those with participant recruitment through AIDS service organization based venues (*P* = 0.02), those with group-based format of delivering interventions (*P* = 0.02), those with retention rate at the latest follow-up <80% (*P* = 0.02), those with a duration of follow-up between 11–15 months (*P* = 0.01), those with mixed risk group participants (*P* = 0.02), those with a sample size at baseline >300 (*P* = 0.01), and those with a rigor score <9 (*P* = 0.01). However, no statistical variation of UVAI with HNUP was found among subgroups in meta-regression (P>0.05). No single study was identified as an outlier in standardized deleted residual analysis in overall meta-analysis and each subgroup of follow-up period. Further sensitivity analyses that were performed by removing the studies using multiple intervention approaches [10], [20], [22] found that the aggregate magnitude of impact of the interventions was weakened after removing the study by Myers et al. [20] (mean ES, −0.10; 95% CI, −0.21, 0.01; *P* = 0.09) (Table 4).

**Table 4:** Subgroup and sensitivity analyses for UVAI with HIV negative or unknown status sexual partners among PLWHA^1^.

Subgroup	No. of intervention	Combined effect size (95% CI)	*P* value	Heterogeneity	
				I^2^ (%)	P value
Recall period on UVAI (months)					
3	8	−0.11 (−0.24, 0.02)	0.11	0	0.65
>3	6	−0.15 (−0.28, −0.02)	0.02	0	0.58
Number of study cities					
1	4	−0.15 (−0.39, 0.09)	0.23	32	0.22
>1	10	−0.12 (−0.23, −0.02)	0.02	0	0.87
Venue of recruiting participants					
AIDS service organizations (ASO)	6	−0.16 (−0.29, −0.03)	0.02	0	0.68
Non-ASO	8	−0.10 (−0.23, 0.03)	0.15	0	0.60
Format of delivering intervention					
Group-based	12	−0.12 (−0.22, −0.02)	0.02	0	0.78
Individual-based	2	−0.23 (−0.58, 0.12)	0.20	27.6	0.24
Duration of follow-up (months)					
Immediately after intervention	3	−0.03 (−0.23, 0.17)	0.78	0	0.95
1–5	4	−0.13 (−0.29, 0.02)	0.09	0	0.46
6–10	12	−0.10 (−0.20, 0.01)	0.08	17.8	0.27
11–15	11	−0.13 (−0.24, −0.03)	0.01	0	0.64
>15	3	−0.03 (−0.25, 0.19)	0.80	0	0.89
Retention rate at the last follow-up					
<80%	7	−0.14 (−0.26, −0.02)	0.02	0	0.81
≥80%	7	−0.11 (−0.25, 0.04)	0.15	0.2	0.42
Risk group					
All MSM	4	−0.11 (−0.28, 0.06)	0.20	0	0.46
Other risk groups or mixed groups	10	−0.13 (−0.24, −0.03)	0.02	0	0.70
Sample size at baseline					
≤300	3	−0.12 (−0.57, 0.33)	0.61	41.8	0.18
>300	11	−0.13 (−0.23, −0.04)	0.01	0	0.85
Rigor score					
<9	9	−0.15 (−0.26, −0.03)	0.01	0	0.68
9	5	−0.10 (−0.25, 0.06)	0.23	0	0.55
Sensitivity analyses					
Excluded Rotheram-Borus et al. [10], 2004 (telephone)	13	−0.13 (−0.23, −0.04)	0.01	0	0.75
Excluded Rotheram-Borus et al. [10], 2004 (in-person)	13	−0.12 (−0.21, −0.03)	0.01	0	0.87
Excluded Myers et al. [20], 2010 (MCP & PS) and (PS)	13	−0.11 (−0.22, −0.01)	0.04	0	0.65
Excluded Myers et al. [20], 2010 (MCP & PS) and (MCP)	13	−0.11 (−0.22, −0.01)	0.04	0	0.65
Excluded Myers et al. [20], 2010 (PS) and (MCP)	13	−0.11 (−0.22, −0.01)	0.03	0	0.64
Excluded Rosser et al. [22], 2010 (Man2Man)	13	−0.14 (−0.23, −0.05)	<0.01	0	0.77
Excluded Rosser et al. [22], 2010 (PoSH)	13	−0.13 (−0.23, −0.04)	0.01	0	0.71

1 UVAI: unprotected vaginal and/or anal intercourse; PLWHA: people living with HIV/AIDS; MSM: men who have sex with men; MCP: medical care provider; PS: prevention specialist; Man2Man: man to man sexual health seminar; PoSH: positive sexual health seminar.

## Discussion

Our systematic meta-analytic review involving 11,286 PLWHA from 15 RCTs studying 18 interventions suggests that prevention interventions are efficacious to reduce UVAI with any sexual partners. This conclusion is robust, as demonstrated in sensitivity analyses by removing some selected studies, which did not alter the findings. Similar meta-analysis was performed separately for UVAI with HIV negative or unknown status sexual partners from 10 RCTs involving 14 interventions, and confirmed the significant efficacy of prevention interventions.

One previous systematic review and meta-analysis of 10 RCTs and two quasi-experimental studies showed that prevention interventions resulted in a 43% overall reduction in unprotected sex, and a 39% reduction in the subgroup analysis of 10 RCTs [15]. Compared with this review, our meta-analysis included eight of these 10 RCTs; two quasi-experimental studies were excluded [51], [52], and two RCTs were also excluded because they did not report total sexual activity (only reporting insertive anal intercourse) [53] or did not provide sufficient information for calculating ES [34]. Our review included 13 additional more recent RCTs [11], [12], [13], [14], [16], [17], [18], [19], [20], [21], [22], [23], [24]; we found that prevention intervention reduced UVAI by 20%. The magnitude of difference in our study was less than that reported in the earlier meta-analysis [15], but it is useful to appreciate the potential impact of these particular approaches in guiding public health investments and designing future research studies.

Our subgroup analyses of trials that assessed outcomes within 15 months post intervention showed a statistically significant relationship between interventions and reduction of UVAI with any sexual partners; while the follow-up assessments from 11 to 15 months showed a non-significant protective trend, but significant protective trend was found after excluding one outlier study [13]. The evidence available for more than 15 months of follow-up intervals was too sparse to draw a conclusion [13], [48]. These results may suggest that the impact of interventions tapers over time, although it is possible that these results merely reflect the dearth of long-term follow-up data. Future studies should evaluate the long-term impact by comparing with and without booster intervention sessions during the follow-up period, though the costs for long-term interventions sometimes are overwhelming for resources-limit settings, and long-term interventions may even not feasible in developed countries, due to manpower or financial or health insurance problems. Governments and researcher might consider developing cost-effective and easy-to-operate intervention sessions to maintain safe sex in long-term time.

Our analyses that stratified by the format of delivering interventions found a 27% reduction of UVAI for group-based interventions, but 14% non-significant reduction for individual-based interventions. This difference by format of delivering interventions was not statistically significant, but the direction of estimates was opposite to one from the previous meta-analysis of studies published between 1988–2004 by Crepaz et al [15], which found that individual-based interventions were more efficacious than group-based (51% vs. 34%). Group-based interventions could be more cost-effective than individual-based; in addition, participants in group-based interventions might be more likely to have opportunities to obtain social support from peers, as shown in studies for treating adult obesity and promoting children's physical activity [54], [55].

The previous review could not assess the impact on UVAI by HIV status of sexual partner [15]. After this review, several RCTs collected this information [11], [12], [18], [20], [21], [22], [23], so that our analysis was able to explore the impact according HIV status of sexual partner for the first time. We found a 13% reduction of UVAI with HNUP in the intervention arms versus the control arms. Most studies were conducted among PLWHA regardless of risk groups, though MSM were accounted for moderate proportion of HIV-positive participants. Only three articles reported UVAI among pure HIV-positive MSM group [12], [22], [50], whereas the effects of interventions targeting pure MSM was not statistically significant. The goal of prevention intervention programs among HIV-infected individuals is to reduce UVAI with those who have not been infected. While this meta-analysis provides valuable preliminary data on the effect on UVAI with HNUP, more studies are needed for assessing effects on UAI and UVI separately, and for investigating the effect within long-term follow-up.

Given the remarkable findings of HPTN 052 that antiretroviral therapy was associated with a significant reduction in HIV transmission [56], [57], perhaps the field should focus on treatment as prevention and the importance of medication adherence. However, sexual risk reduction should also be a component of combined intervention packages in future intervention programs. By combining results across trials we saw a significant average benefit of prevention interventions on unprotected sex, whereas most of the individual trials failed to show a statistically significant benefit. This suggests that many of these studies were underpowered. Surprisingly, although these were trials of different prevention interventions in different patient populations, there was no evidence to suggest cross-study heterogeneity in outcomes. Although this is not evidence that all prevention interventions are universally good, it does support the idea that several modestly efficacious prevention intervention options are available and that we lack strong evidence that one is better than another. The reasons for differential success may be due less to the content of the interventions and more to design issues (e.g., content of the control group).

Several issues related to the original studies require commentary. The primary outcome UVAI was self-reported; and therefore, it may be subject to social desirability bias. In addition, even though thirteen international databases were explored, all included RCTs were conducted in USA; three RCTs in Africa were excluded because no target outcomes were reported [58], [59] or not enough data were available for calculation [36]. We may not be able to extrapolate the findings from US-based trials to other cultural settings. Therefore, future clinical trials of risk reduction techniques outside of the United States are needed. Our meta-analysis has its own limitations. First, our meta-analysis only included published RCTs. We did not make requests to the scientific community (e.g., listserv) and/or to individual authors with relevant research, so unpublished or in print RCTs were not included. In addition, four trials were excluded because we did not get needed data for analysis from the investigators [34], [35], [36], [37]. Second, non-English databases were not included in our systematic searching; therefore, data from papers published in other languages might have been missed. Excluding non-English publications may generally have little effect on meta-analysis outcomes, but it is difficult to predict the importance of non-English language trials for individual systematic reviews [60]. Third, some trials had multiple interventions compared against a single control condition, and the outcomes from these trials were treated as separate findings in the meta-analysis, perhaps giving undue weights to these multi-intervention trials. However, sensitivity analyses revealed stable overall trends even when these trials were excluded. Finally, we only included RCTs; three quasi-experimental studies were excluded [51], [52], [61], because this design might overestimate intervention efficacy based on previous meta-analytic review [15].

Strengths of our approach are also worthy of mention. We adjusted for baseline differences among intervention groups, we combined continuous and categorical outcomes, and we assessed the impact on UVAI by HIV serostatus of sexual partners. The evaluation for HNUP provides additional information for developing public health programs. In summary, our meta-analysis suggests that prevention interventions are efficacious, particularly in the short-term, at reducing UVAI. Positive prevention approaches should be included in HIV prevention programs even as the research community seeks to improve their efficacy. Booster intervention sessions may be needed to achieve long-term impact on reducing unprotected sex among PLWHA in future risk reduction programs.

## Supporting Information

File S1 **69 excluded studies and exclusion reasons.**
(DOCX)

Checklist S1 **PRISMA Checklist.**
(DOC)
