# Supplementary material for: Sexual Risk Reduction for HIV-Infected Persons: A Meta-Analytic Review of “Positive Prevention” Randomized Clinical Trials
Source: PLoS One. 2014 Sep 22;9(9):e107652. doi: 10.1371/journal.pone.0107652 (PMC4171502; doi:10.1371/journal.pone.0107652)
Supplement: File S1 — 69 excluded studies and exclusion reasons. (DOCX) [file pone.0107652.s001.docx]

**File S1:** 69 excluded studies and exclusion reasons

*(A)* *Not original articles but editorials, comments or reviews*

1. Crepaz N, Lyles CM, Wolitski RJ, Passin WF, Rama SM, Herbst JH, et al. Do prevention interventions reduce HIV risk behaviours among people living with HIV? A meta-analytic review of controlled trials. AIDS 2006;20(2):143-57.
2. Johnson BT, Carey MP, Chaudoir SR, Reid AE. Sexual risk reduction for persons living with HIV: research synthesis of randomized controlled trials, 1993 to 2004. J Acquir Immune Defic Syndr 2006;41(5):642-50.
3. Herbst JH, Kay LS, Passin WF, Lyles CM, Crepaz N, Marin BV. A systematic review and meta-analysis of behavioral interventions to reduce HIV risk behaviors of Hispanics in the United States and Puerto Rico. AIDS and Behavior 2007;11(1):25-47.
4. Kennedy CE, Medley AM, Sweat MD, O'Reilly KR. Behavioural interventions for HIV positive prevention in developing countries: a systematic review and meta-analysis. Bull World Health Organ 2010;88(8):615-23.
5. Carvalho FT, Goncalves TR, Faria ER, Shoveller JA, Piccinini CA, Ramos MC, et al. Behavioral interventions to promote condom use among women living with HIV. Cochrane Database Syst Rev 2011;9:CD007844.

*(B) No information on target outcomes or measures of interest*

1. Kelly JA, Murphy DA, Bahr GR, Kalichman SC, Morgan MG, Stevenson LY, et al. Outcome of cognitive-behavioral and support group brief therapies for depressed, HIV-infected persons. Am J Psychiatry 1993;150(11):1679-86.
2. Wagner GJ, Rabkin JG, Rabkin R. Sexual activity among HIV-seropositive gay men seeking treatment for depression. J Clin Psychiatry 1993;54(12):470-5.
3. MacNeil JM, Mberesero F, Kilonzo G. Is care and support associated with preventive behaviour among people with HIV? AIDS care 1999;11(5):537-46.
4. Wight RG, Rotheram-Borus MJ, Klosinski L, Ramos B, Calabro M, Smith R. Screening for transmission behaviors among HIV-infected adults. AIDS Educ Prev 2000;12(5):431-41.
5. Fogarty LA, Heilig CM, Armstrong K, Cabral R, Galavotti C, Gielen AC, et al. Long-term effectiveness of a peer-based intervention to promote condom and contraceptive use among HIV-positive and at-risk women. Public Health Rep 2001;116 Suppl 1:103-19.
6. Jones DL, Weiss SM, Malow R, Ishii M, Devieux J, Stanley H, et al. A brief sexual barrier intervention for women living with AIDS: acceptability, use, and ethnicity. J Urban Health 2001;78(4):593-604.
7. Patterson TL, Shaw WS, Semple SJ. Reducing the sexual risk behaviors of HIV+ individuals: outcome of a randomized controlled trial. Ann Behav Med 2003;25(2):137-45.
8. Wyatt GE, Longshore D, Chin D, Carmona JV, Loeb TB, Myers HF, et al. The efficacy of an integrated risk reduction intervention for HIV-positive women with child sexual abuse histories. AIDS Behav 2004;8(4):453-62.
9. Gore-Felton C, Rotheram-Borus MJ, Weinhardt LS, Kelly JA, Lightfoot M, Kirshenbaum SB, et al. The Healthy Living Project: an individually tailored, multidimensional intervention for HIV-infected persons. AIDS Educ Prev 2005;17(1 Suppl A):21-39.
10. Jones DL, Ross D, Weiss SM, Bhat G, Chitalu N. Influence of partner participation on sexual risk behavior reduction among HIV-positive Zambian women. J Urban Health 2005;82(3 Suppl 4):iv92-100.
11. Kalichman SC, Rompa D, Cage M. Group intervention to reduce HIV transmission risk behavior among persons living with HIV/AIDS. Behav Modif 2005;29(2):256-85.
12. Jones DL, Weiss SM, Bhat GJ, Bwalya V. Influencing sexual practices among HIV-positive Zambian women. AIDS Care 2006;18(6):629-34.
13. Kalichman SC, Cherry C, Cain D, Pope H, Kalichman M, Eaton L, et al. Internet-based health information consumer skills intervention for people living with HIV/AIDS. J Consult Clin Psychol 2006;74(3):545-54.
14. Johnson MO, Charlebois E, Morin SF, Remien RH, Chesney MA. Effects of a behavioral intervention on antiretroviral medication adherence among people living with HIV: the healthy living project randomized controlled study. J Acquir Immune Defic Syndr 2007;46(5):574-80.
15. Morin SF, The Healthy Living Project Team. Effects of a behavioral intervention to reduce risk of transmission among people living with HIV: the healthy living project randomized controlled study. J Acquir Immune Defic Syndr 2007;44(2):213-21.
16. Cornman DH, Kiene SM, Christie S, Fisher WA, Shuper PA, Pillay S, et al. Clinic-based intervention reduces unprotected sexual behavior among HIV-infected patients in KwaZulu-Natal, South Africa: results of a pilot study. J Acquir Immune Defic Syndr 2008;48(5):553-60.
17. Maisto SA, Conigliaro JC, Gordon AJ, McGinnis KA, Justice AC. An experimental study of the agreement of self-administration and telephone administration of the Timeline Followback interview. J Stud Alcohol Drugs 2008;69(3):468-71.
18. Morin SF, Shade SB, Steward WT, Carrico AW, Remien RH, Rotheram-Borus MJ, et al. A behavioral intervention reduces HIV transmission risk by promoting sustained serosorting practices among HIV-infected men who have sex with men. J Acquir Immune Defic Syndr 2008;49(5):544-51.
19. Carrico AW, Chesney MA, Johnson MO, Morin SF, Neilands TB, Remien RH, et al. Randomized controlled trial of a cognitive-behavioral intervention for HIV-positive persons: an investigation of treatment effects on psychosocial adjustment. AIDS Behav 2009;13(3):555-63.
20. Coleman CL, Jemmott L, Jemmott JB, Strumpf N, Ratcliffe S. Development of an HIV risk reduction intervention for older seropositive African American men. AIDS Patient Care STDS 2009;23(8):647-55.
21. Naar-King S, Parsons JT, Murphy DA, Chen X, Harris DR, Belzer ME. Improving health outcomes for youth living with the human immunodeficiency virus: a multisite randomized trial of a motivational intervention targeting multiple risk behaviors. Arch Pediatr Adolesc Med 2009;163(12):1092-8.
22. Saleh-Onoya D, Reddy PS, Ruiter RA, Sifunda S, Wingood G, van den Borne B. Condom use promotion among isiXhosa speaking women living with HIV in the Western Cape Province, South Africa: a pilot study. AIDS Care 2009;21(7):817-25.
23. Serovich JM, Reed S, Grafsky EL, Andrist D. An intervention to assist men who have sex with men disclose their serostatus to casual sex partners: results from a pilot study. AIDS Educ Prev 2009;21(3):207-19.
24. Velasquez MM, von Sternberg K, Johnson DH, Green C, Carbonari JP, Parsons JT. Reducing sexual risk behaviors and alcohol use among HIV-positive men who have sex with men: a randomized clinical trial. J Consulting Clin Psychol 2009;77(4):657-67.
25. Cosio D, Heckman TG, Anderson T, Heckman BD, Garske J, McCarthy J. Telephone-administered motivational interviewing to reduce risky sexual behavior in HIV-infected rural persons: a pilot randomized clinical trial. Sex Transm Dis 2010;37(3):140-6.
26. Li L, Lee SJ, Jiraphongsa C, Khumtong S, Iamsirithaworn S, Thammawijaya P, et al. Improving the health and mental health of people living with HIV/AIDS: 12-month assessment of a behavioral intervention in Thailand. Am J Public Health 2010;100(12):2418-25.
27. Naar-King S, Parsons JT, Murphy D, Kolmodin K, Harris DR. A multisite randomized trial of a motivational intervention targeting multiple risks in youth living with HIV: initial effects on motivation, self-efficacy, and depression. J Adolesc Health 2010;46(5):422-8.
28. Chen X, Murphy DA, Naar-King S, Parsons JT. A clinic-based motivational intervention improves condom use among subgroups of youth living with HIV. J Adolesc Health 2011;49(2):193-8.
29. Holstad MM, DiIorio C, Kelley ME, Resnicow K, Sharma S. Group motivational interviewing to promote adherence to antiretroviral medications and risk reduction behaviors in HIV infected women. AIDS Behav 2011;15(5):885-96.
30. Kalichman SC, Cherry C, Kalichman MO, Amaral CM, White D, Pope H, et al. Integrated behavioral intervention to improve HIV/AIDS treatment adherence and reduce HIV transmission. Am J Public Health 2011;101(3):531-8.
31. Puffer ES, Kochman A, Hansen NB, Sikkema KJ. An evidence-based group coping intervention for women living with HIV and history of childhood sexual abuse. Int J Group Psychother 2011;61(1):98-126.

*(C) Not randomized clinical trials*

1. Semple SJ, Patterson TL, Grant I. Psychosocial predictors of unprotected anal intercourse in a sample of HIV positive gay men who volunteer for a sexual risk reduction intervention. AIDS Educ Prev 2000;12(5):416-30.
2. Grinstead O, Zack B, Faigeles B. Reducing postrelease risk behavior among HIV seropositive prison inmates: the health promotion program. AIDS Educ Prev 2001;13(2):109-19.
3. Rotheram-Borus MJ, Lee MB, Murphy DA, Futterman D, Duan N, Birnbaum JM, et al. Efficacy of a preventive intervention for youths living with HIV. Am J Public Health 2001;91(3):400-5.
4. Amirkhanian YA, Kelly JA, McAuliffe TL. Psychosocial needs, mental health, and HIV transmission risk behavior among people living with HIV/AIDS in St Petersburg, Russia. AIDS 2003;17(16):2367-74.
5. Halkitis PN, Green KA, Remien RH, Stirratt MJ, Hoff CC, Wolitski RJ, et al. Seroconcordant sexual partnerings of HIV-seropositive men who have sex with men. AIDS 2005;19 (Suppl 1):S77-86.
6. Halkitis PN, Wilton L, Wolitski RJ, Parsons JT, Hoff CC, Bimbi DS. Barebacking identity among HIV-positive gay and bisexual men: demographic, psychological, and behavioral correlates. AIDS 2005;19 (Suppl 1):S27-35.
7. Ibanez GE, Purcell DW, Stall R, Parsons JT, Gomez CA. Sexual risk, substance use, and psychological distress in HIV-positive gay and bisexual men who also inject drugs. AIDS 2005;19 (Suppl 1):S49-55.
8. O'Leary A, Wolitski RJ, Remien RH, Woods WJ, Parsons JT, Moss S, et al. Psychosocial correlates of transmission risk behavior among HIV-seropositive gay and bisexual men. AIDS 2005;19 (Suppl 1):S67-75.
9. Purcell DW, Moss S, Remien RH, Woods WJ, Parsons JT. Illicit substance use, sexual risk, and HIV-positive gay and bisexual men: differences by serostatus of casual partners. AIDS 2005;19 (Suppl 1):S37-47.
10. Bunnell R, Ekwaru JP, Solberg P, Wamai N, Bikaako-Kajura W, Were W, et al. Changes in sexual behavior and risk of HIV transmission after antiretroviral therapy and prevention interventions in rural Uganda. AIDS 2006;20(1):85-92.
11. da Silveira MF, dos Santos IS. Impact of an educational intervention to promote condom use among the male partners of HIV positive women. J Eval Clin Pract 2006;12(1):102-11.
12. Rice E, Batterham P, Rotheram-Borus MJ. Unprotected sex among youth living with HIV before and after the advent of highly active antiretroviral therapy. Perspect Sex Reprod Health 2006;38(3):162-7.
13. Pearson CR, Kurth AE, Cassels S, Martin DP, Simoni JM, Hoff P, et al. Modeling HIV transmission risk among Mozambicans prior to their initiating highly active antiretroviral therapy. AIDS Care 2007;19(5):594-604.
14. Luchters S, Sarna A, Geibel S, Chersich MF, Munyao P, Kaai S, et al. Safer sexual behaviors after 12 months of antiretroviral treatment in Mombasa, Kenya: a prospective cohort. AIDS Patient Care STDS 2008;22(7):587-94.
15. Cheng WS, Garfein RS, Semple SJ, Strathdee SA, Zians JK, Patterson TL. Differences in sexual risk behaviors among male and female HIV-seronegative heterosexual methamphetamine users. Am J Drug Alcohol Abuse 2009;35(5):295-300.
16. Coleman SM, Rajabiun S, Cabral HJ, Bradford JB, Tobias CR. Sexual risk behavior and behavior change among persons newly diagnosed with HIV: the impact of targeted outreach interventions among hard-to-reach populations. AIDS Patient Care STDS 2009;23(8):639-45.
17. Spikes PS, Purcell DW, Williams KM, Chen Y, Ding H, Sullivan PS. Sexual risk behaviors among HIV-positive black men who have sex with women, with men, or with men and women: implications for intervention development. Am J Public Health 2009;99(6):1072-8.
18. Welles SL, Baker AC, Miner MH, Brennan DJ, Jacoby S, Rosser BR. History of childhood sexual abuse and unsafe anal intercourse in a 6-city study of HIV-positive men who have sex with men. Am J Public Health 2009;99(6):1079-86.
19. Barta WD, Tennen H, Kiene SM. Alcohol-involved sexual risk behavior among heavy drinkers living with HIV/AIDS: negative affect, self-efficacy, and sexual craving. Psychol Addict Behav 2010;24(4):563-70.
20. Peltzer K, Tabane C, Matseke G, Simbayi L. Lay counsellor-based risk reduction intervention with HIV positive diagnosed patients at public HIV counselling and testing sites in Mpumalanga, South Africa. Eval Program Plann 2010;33(4):379-85.
21. Copenhaver MM, Lee IC, Margolin A, Bruce RD, Altice FL. Testing an optimized community-based human immunodeficiency virus (HIV) risk reduction and antiretroviral adherence intervention for HIV-infected injection drug users. Subst Abus 2011;32(1):16-26.
22. Heitgerd JL, Kalayil EJ, Patel-Larson A, Uhl G, Williams WO, Griffin T, et al. Reduced sexual risk behaviors among people living with HIV: Results from the Healthy Relationships Outcome Monitoring Project. AIDS Behav 2011;15(8):1677-90.
23. Safren SA, O'Cleirigh C, Skeer MR, Driskell J, Goshe BM, Covahey C, et al. Demonstration and evaluation of a peer-delivered, individually-tailored, HIV prevention intervention for HIV-infected MSM in their primary care setting. AIDS Behav 2011;15(5):949-58.

*(D) HIV-negative study subjects included*

1. Kamenga M, Ryder RW, Jingu M, Mbuyi N, Mbu L, Behets F, et al. Evidence of marked sexual behavior change associated with low HIV-1 seroconversion in 149 married couples with discordant HIV-1 serostatus: experience at an HIV counselling center in Zaire. AIDS 1991;5(1):61-7.
2. Allen S, Tice J, Van de Perre P, Serufilira A, Hudes E, Nsengumuremyi F, et al. Effect of serotesting with counselling on condom use and seroconversion among HIV discordant couples in Africa. BMJ 1992;304(6842):1605-9.
3. van der Straten A, Gomez CA, Saul J, Quan J, Padian N. Sexual risk behaviors among heterosexual HIV serodiscordant couples in the era of post-exposure prevention and viral suppressive therapy. AIDS 2000;14(4):F47-54.
4. Dushay RA, Singer M, Weeks MR, Rohena L, Gruber R. Lowering HIV risk among ethnic minority drug users: comparing culturally targeted intervention to a standard intervention. Am J Drug Alcohol Abuse 2001;27(3):501-24.
5. Yang F, Wu Z, Xu C. Acceptability and feasibility of promoting condom use among families with human immunodeficiency virus infection in rural area of China. Zhonghua Liu Xing Bing Xue Za Zhi 2001;22(5):330-3. [Chinese].
6. Carey MP, Carey KB, Maisto SA, Gordon CM, Schroder KE, Vanable PA. Reducing HIV-risk behavior among adults receiving outpatient psychiatric treatment: results from a randomized controlled trial. J Consult Clin Psychol 2004;72(2):252-68.
7. El-Bassel N, Jemmott JB, Landis JR, Pequegnat W, Wingood GM, Wyatt GE, et al. National Institute of Mental Health Multisite Eban HIV/STD Prevention Intervention for African American HIV Serodiscordant Couples: a cluster randomized trial. Arch Intern Med 2010;170(17):1594-601.
8. Tieu HV, Xu G, Bonner S, Spikes P, Egan JE, Goodman K, et al. Sexual partner characteristics, serodiscordant/serostatus unknown unprotected anal intercourse and disclosure among human immunodeficiency virus-infected and uninfected black men who have sex with men in New York City. Sex Transm Dis 2011;38(6):548-54.

*(E) Repeated reporting of the same study*

1. Purcell DW, Metsch LR, Latka M, Santibanez S, Gomez CA, Eldred L, et al. Interventions for seropositive injectors-research and evaluation: an integrated behavioral intervention with HIV-positive injection drug users to address medical care, adherence, and risk reduction. J Acquir Immune Defic Syndr 2004;37 (Suppl 2):S110-8.
2. Milam J, Richardson JL, McCutchan A, Stoyanoff S, Weiss J, Kemper C, et al. Effect of a brief antiretroviral adherence intervention delivered by HIV care providers. J Acquir Immune Defic Syndr 2005;40(3):356-63.
3. O'Leary A, Hoff CC, Purcell DW, Gomez CA, Parsons JT, Hardnett F, et al. What happened in the SUMIT trial? Mediation and behavior change. AIDS 2005;19 (Suppl 1):S111-21.
4. Hoff CC, Pals SL, Purcell DW, Parsons JT, Halkitis PN, Remien RH, et al. Examining the role of partner status in an HIV prevention trial targeting HIV-positive gay and bisexual men: the seropositive urban men's intervention trial (SUMIT). AIDS Behav 2006;10(6):637-48.
5. Kidder DP, Wolitski RJ, Royal S, Aidala A, Courtenay-Quirk C, Holtgrave DR, et al. Access to housing as a structural intervention for homeless and unstably housed people living with HIV: rationale, methods, and implementation of the housing and health study. AIDS Behav 2007;11(6 Suppl):149-61.
6. Rotheram-Borus MJ, Desmond K, Comulada WS, Arnold EM, Johnson M. Reducing risky sexual behavior and substance use among currently and formerly homeless adults living with HIV. Am J Public Health 2009;99(6):1100-7.
